# Causal ALS genes impact the MHC class II antigen presentation pathway

**DOI:** 10.1073/pnas.2305756120

**Published:** 2023-09-18

**Authors:** Binkai Chi, Muhammet M. Öztürk, Christina L. Paraggio, Claudia E. Leonard, Maria E. Sanita, Mahtab Dastpak, Jeremy D. O’Connell, Jordan A. Coady, Jiuchun Zhang, Steven P. Gygi, Rodrigo Lopez-Gonzalez, Shanye Yin, Robin Reed

**Affiliations:** ^a^Department of Cell Biology, Blavatnik Institute, Harvard Medical School, Boston, MA 02115; ^b^Harvard Medical School Cell Biology Initiative for Genome Editing and Neurodegeneration, Blavatnik Institute, Harvard Medical School, Boston, MA 02115; ^c^Department of Neurosciences Lerner Research Institute, Cleveland Clinic, Cleveland, OH 44196; ^d^Department of Pathology, Albert Einstein College of Medicine, Bronx, NY 10461

**Keywords:** ALS, FUS, C9ORF72, MHC II

## Abstract

Mutations in RNA/DNA-binding proteins cause amyotrophic lateral sclerosis (ALS), but the underlying disease mechanisms remain unclear. Here, we report that a set of ALS-associated proteins, namely FUS, EWSR1, TAF15, and MATR3, impact the expression of genes encoding the major histocompatibility complex II (MHC II) antigen presentation pathway. Both subunits of the MHC II heterodimer, HLA-DR, are down-regulated in ALS gene knockouts/knockdown in HeLa and human microglial cells, due to loss of the MHC II transcription factor CIITA. Importantly, hematopoietic progenitor cells (HPCs) derived from human embryonic stem cells bearing the FUS^R495X^ mutation and HPCs derived from C9ORF72 ALS patient induced pluripotent stem cells also exhibit disrupted MHC II expression. Given that HPCs give rise to numerous immune cells, our data raise the possibility that loss of the MHC II pathway results in global failure of the immune system to protect motor neurons from damage that leads to ALS.

## Significance

Amyotrophic lateral sclerosis (ALS) is a devastating neurodegenerative disease characterized by loss of motor neurons. Mutations in over 30 genes can cause ALS, but the underlying mechanism(s) remains unknown. In this work, we demonstrate that five ALS genes (FUS, EWSR1, TAF15, MATR3, and C9ORF72) regulate gene expression of the MHC (major histocompatibility complex) class II antigen-presenting pathway. ALS-causative mutations in FUS and C9ORF72 lead to significant downregulation of the MHC II pathway in hematopoietic progenitor cells, the cell type that gives rise to a variety of immune cells. Our findings suggest that downregulation of the MHC II pathway in immune cells is linked to ALS and is a potential therapeutic target.

Amyotrophic lateral sclerosis (ALS) is a progressive fatal motor neuron disease with no effective treatment. Familial ALS is caused by mutations in ~30 genes, which function in diverse cellular pathways (1–3). These pathways include protein homeostasis, gene expression, mitochondrial function, intracellular transport, endoplasmic reticulum stress, RNA splicing, and several others (1–3). Thus, a major challenge in ALS is to determine how and when the disrupted pathways contribute to the progression of ALS, as different pathways are known or are likely to contribute at different stages of disease progression (4–8). ALS is even more complex as, although motor neurons are the cell type directly impacted, supporting cells in the central nervous system (CNS), such as microglia, oligodendrocytes, and astrocytes also play critical roles (9–11). Unraveling the contributions of these glial cells to ALS is a key challenge, with models positing both detrimental and protective roles (12–17). Furthermore, many types of systemic immune cells throughout the body play important roles in ALS by, for example, migrating into the CNS and secreting cytokines or neurotrophic factors that can be proinflammatory or anti-inflammatory (18–24). The data suggest that immune cells are protective early in the disease course but then become toxic as the disease progresses, accompanied by the contribution of proinflammatory cytokines to disease advancement (17).

Among the important insights into ALS disease mechanisms was the observation that >1/3 of ALS genes encode RNA/DNA-binding proteins (25), most of which have known roles in gene expression. We recently characterized the proteome of a cellular complex we termed the RNAP polymerase II (RNAP II)/U1 snRNP machinery (26), and we identified that this machinery is a central hub for most of the ALS-associated RNA/DNA-binding proteins. The machinery also includes several spinal muscular atrophy (SMA)-causative proteins, and these also play roles in gene expression (26). In prior studies, we provided evidence that ALS and SMA are linked to one another at the molecular level through direct interactions of proteins involved in each disease (27, 28). Thus, the finding that ALS/SMA proteins share the same machinery is not surprising considering that numerous steps in gene expression are extensively coupled both physically and functionally (29–31). The coupling involves virtually all steps in gene expression including transcription, capping, splicing, 3′ end formation, mRNA export, and mRNA degradation, and proteins that function in these coupled processes are components of the RNAP II/U1 snRNP machinery (26). Thus, a critical question is whether the ALS proteins associated with this machinery have downstream roles in different cellular pathways or converge on a common pathway(s).

Among the ALS genes in the RNAP II/U1 snRNP machinery are FUS (Fused in Sarcoma), TAF15 (TATA-box binding protein Associated Factor 15), and MATR3 (Matrin 3) (26). These proteins are structurally similar to one another, and to EWSR1 (Ewing Sarcoma breakpoint region 1/EWS RNA binding protein 1), a putative ALS protein (32). FUS, EWSR1, and TAF15 comprise the FET family of proteins which were discovered based on their roles in transcription, as it was found that the transcription activation domains of the FET family members translocate to other genes to generate potent oncogenic fusion proteins (33–35). More recently, FET family members were reported to play roles in other steps of gene expression, such as RNA splicing, though these roles may be indirect via coupling of transcription to splicing and other gene expression steps (32).

To investigate the convergent functions of this group of ALS proteins in gene expression, we examined the global gene expression of the CRISPR-knockout (KO) lines of the FET family members and MATR3 using mass spectrometry. Strikingly, we found that the MHC II (Major Histocompatibility Complex class II) antigen presentation pathway was strongly down-regulated in FUS, TAF15, and MATR3 KOs. Furthermore, we demonstrated that loss of the MHC II pathway in the ALS KOs was due to downregulation of CIITA (Class II Major Histocompatibility Complex Transactivator), the master transcriptional regulator of this pathway. Importantly, the MHC II pathway was down-regulated in the HMC3 human microglial cell line and hematopoietic progenitor cells (HPCs) bearing an ALS mutation in FUS, providing evidence for a direct link between ALS and the MHC II pathway. Moreover, we also observed this phenotype in HPCs carrying the ALS-causative C9ORF72 GGGGCC hexanucleotide repeat expansion (HRE), which is the most frequent genetic cause of ALS. Indeed, the MHC II pathway may be highly pertinent to ALS because it is essential in all MHC II cells, which are a crucial component of the immune system, playing key roles both directly in the CNS and indirectly via the systemic circulatory system. Collectively, our data are consistent with a model in which a subset of ALS genes regulates the MHC II pathway through CIITA. Our data further suggest that CIITA, with its tight restriction to the MHC II pathway, as well as the other genes in the MHC II pathway, may be therapeutic targets for multiple forms of ALS.

## Results and Discussion

### Multiple ALS Genes Regulate Expression of Immune Response Pathways.

**Fig. 1.**
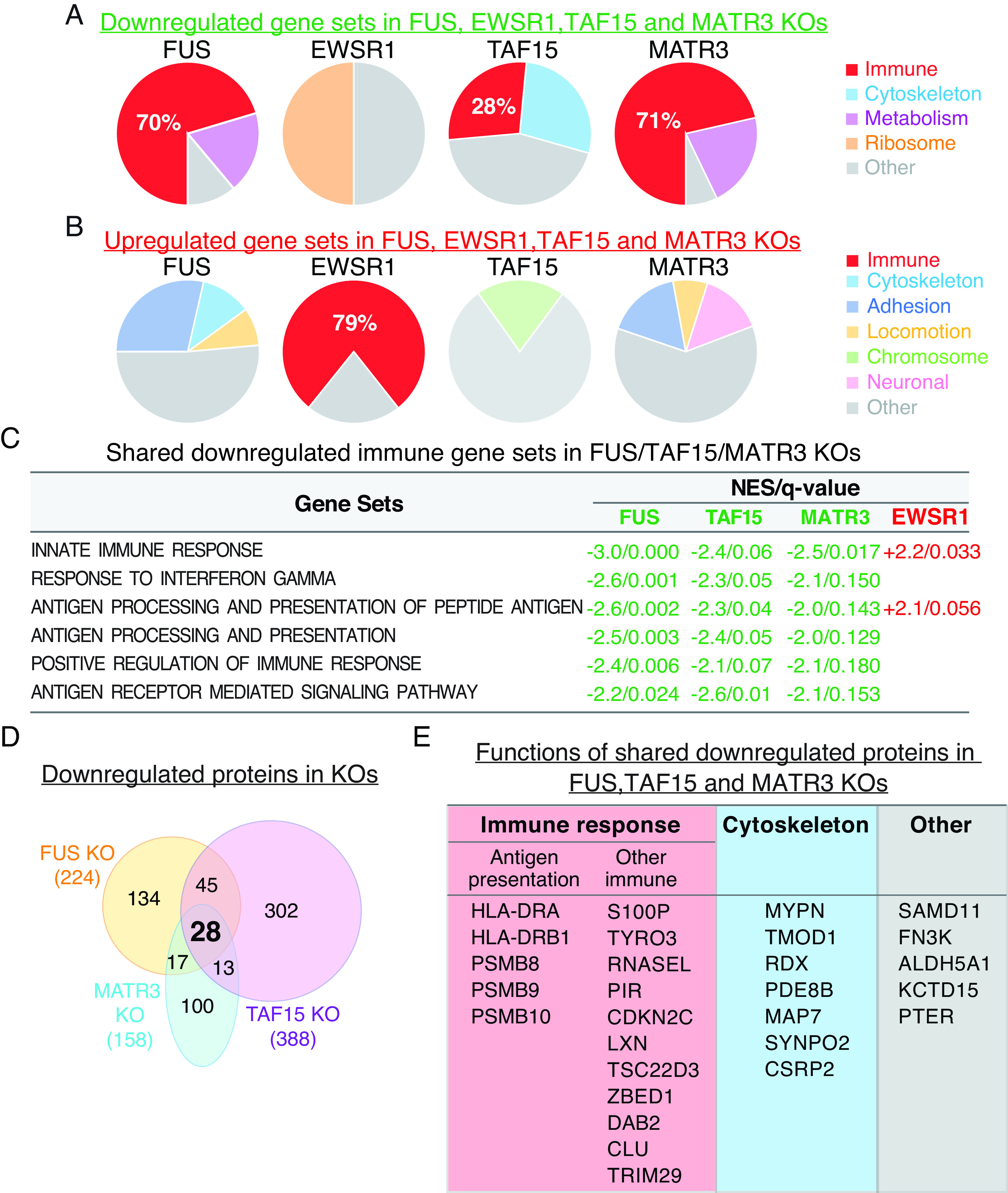
Immune gene sets are top down-regulated in FUS, TAF15, and MATR3 ALS gene KO HeLa cells. (*A*) Pie charts showing the percentage of down-regulated immune gene sets in red in each KO. (*B*) Immune gene sets (shown in red) are top up-regulated in EWSR1 KO. Other categories of down-regulated gene sets in each KO are indicated. (*C*) Immune gene sets shared among FUS, TAF15, and MATR3 KO lines. Green and red font shows Nominal Enrichment Score (NES) of down- or up-regulated gene sets, respectively. q-value indicates false discovery rate (FDR). (*D*) Venn diagram showing the 28 shared down-regulated proteins in FUS, TAF15, and MATR3 KOs. (*E*) List of the 28 shared proteins with their general functions.

To investigate the functions of the ALS-associated FET family members and MATR3, we used quantitative mass spectrometry to analyze protein expression in our previously established 4 KO HeLa lines (28) as loss-of-function effects of these proteins have been implicated in ALS pathogenesis (3, 25, 36–38). Two independent clones were used for each KO. Mass spectrometry identified over 6,000 proteins (Dataset S1). Examination of the significantly dysregulated proteins (*P*-value < 0.05, Dataset S2) by Gene Set Enrichment Analysis (GSEA) revealed striking differences in the down-regulated vs. up-regulated gene sets (Datasets S3 and S4, respectively) in the FUS, TAF15, and MATR3 KOs. Immune-related gene sets were selectively highly enriched in the down-regulated (Fig. 1*A*) but not in the up-regulated group (Fig. 1*B*). For example, among the down-regulated gene sets identified in FUS KO, about 70% are immune-related. Six of the immune gene sets in the down-regulated group were shared among the FUS, TAF15, and MATR3 KOs, and 2 of these were up-regulated in EWSR1 KO (Fig. 1*C*). The observation that multiple immune gene sets are shared in the down-regulated group of FUS, TAF15 and MATR3 KOs raises the possibility that these 3 ALS proteins impact the disease via downregulation of immune pathways.

We next analyzed the data at the level of individual proteins (Fig. 1*D*). Among the significantly down-regulated proteins in the FUS, TAF15, and MATR3 KOs, we identified 28 proteins that displayed decreased expression in all 3 lines (Fig. 1*D* and Dataset S5). Among these, more than half play roles in the innate immune response, aligning with the GSEA results (Fig. 1*E*). Notably, we observed downregulation of multiple proteins that function in antigen presentation in all 3 KOs (Fig. 1*E*). These include HLA-DRA (Human leukocyte antigen II histocompatibility antigen, DR alpha) and HLA-DRB1 (Human leukocyte antigen II histocompatibility antigen, DR beta 1), which play key roles in MHC class II antigen presentation (39, 40) (see below). In addition, all 3 immunoproteasome-specific proteases, PSMBs 8-10, which function in MHC class I antigen presentation (41–43) were down-regulated. Other down-regulated proteins involved in antigen presentation include DAB2 and CLU (44, 45), both of which play roles in phagocytosis (Fig. 1*E*). Finally, additional proteins that function in immunity were down-regulated, such as RNASEL and S100P (Fig. 1*E*) (46, 47). We did not detect alterations in the expression level of TDP-43, a protein commonly associated with ALS pathology, in any of the KOs (Dataset S1). Together, these data indicate that FUS, TAF15, and MATR3 each play a key role in regulating the immune response.

### Regulation of MHC II Antigen Presentation Genes by FUS, TAF15, and MATR3.

**Fig. 2.**
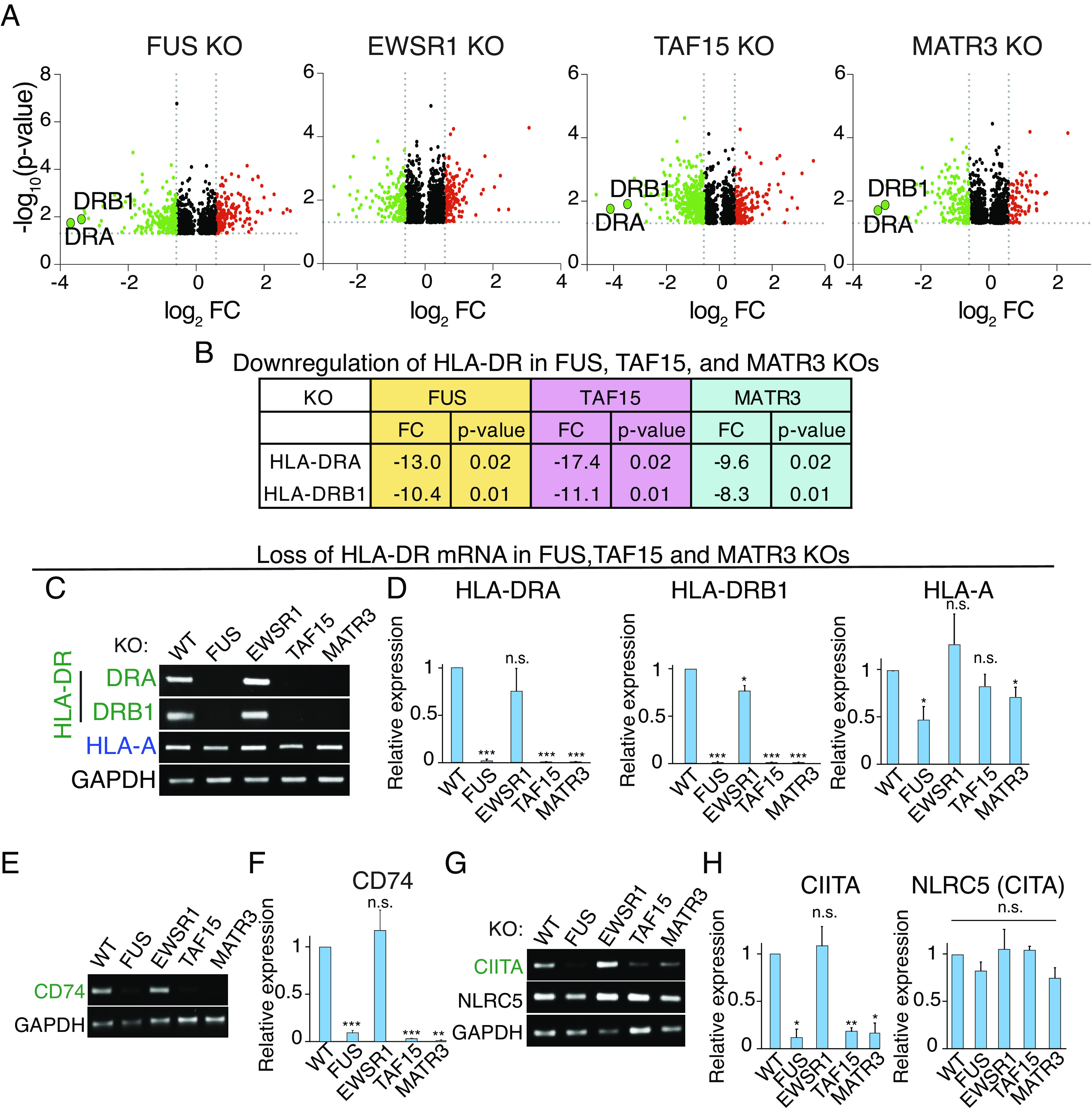
Downregulation of MHC II pathway genes in FUS, TAF15, and MATR3 KO HeLa cells. (*A*) Volcano plots of proteins in HeLa KO lines. HLA-DRA and -DRB1 are indicated. (*B*) Fold change and *P*-values of down-regulated HLA-DRA and -DRB1 in the indicated KO lines. (*C* and *D*) RT-PCR (*C*) and qPCR (*D*) from total RNA isolated from the indicated WT and KO lines for the HLA mRNAs shown. GAPDH was used as an internal control. *P < 0.05. **P < 0.01. ***P < 0.001. n.s., nonsignificant (paired Student’s *t* test). Error bars represent SD. (*E* and *F*) Same as (*C* and *D*), except CD74 was examined. (*G* and *H*) Same as (*C* and *D*), except that CIITA and NLRC5 were examined.

The most striking of the down-regulated immune proteins were HLA-DRA and HLA-DRB1, which were the top down-regulated proteins in all 3 KOs (Fig. 2*A*) but unchanged in the EWSR1 KO (Dataset S5). HLA-DRA and HLA-DRB1 comprise the HLA-DR heterodimer, which is a cell surface receptor that presents antigens to T cells. These interactions initiate a wide range of immune responses including the production of cytokines and antibodies (39, 48, 49). The observation that both components of the heterodimer are down-regulated and to such a great extent in the 3 KOs (~10 fold down, Fig. 2*B*) strongly indicates that HLA-DR regulation is an important function of FUS, TAF15, and MATR3 that has not been previously recognized.

To investigate whether mRNA levels of HLA-DRA and HLA-DRB1 were affected in the KOs, we carried out RT-PCR using total RNA from the wild type (WT) and KO HeLa lines. As shown in Fig. 2*C*, HLA-DRA and HLA-DRB1 mRNA levels were strongly reduced in FUS, TAF15, and MATR3 KOs but unaffected in the EWSR1 KO. In contrast, mRNA levels of MHC class I gene HLA-A, were not significantly affected in any of the KOs (Fig. 2*C*). These results were confirmed by qPCR (Fig. 2*D*).

Based on our results with the HLA-DR heterodimer, we further examined the MHC II pathway. Specifically, RT-PCR revealed that CD74 mRNA levels were strongly decreased in the FUS, TAF15, and MATR3 KOs but not affected in the EWSR1 KO (Fig. 2*E*), and qPCR confirmed these results (Fig. 2*F*). This is particularly noteworthy as CD74, which was previously known as the ‘HLA-DR gamma chain’, plays a critical role in MHC II antigen presentation by stabilizing HLA-DR immediately after its synthesis and then chaperoning it to the endosomal system for antigen processing (40, 50). We conclude that 3 of the key components for MHC II antigen presentation pathway are drastically down-regulated at the mRNA level in FUS, TAF15, and MATR3 KOs.

We next investigated mechanisms for downregulation of the HLA-DR heterodimer and CD74 in the KO lines. FUS, TAF15, and MATR3 have known roles in transcription (33, 34, 51), and we did not detect any splicing defects in the MHC II mRNAs. This result was not unexpected, as different splicing factors recognize distinct sequence elements within pre-mRNAs and are therefore highly unlikely to missplice the same pre-mRNAs. Thus, we next examined expression of the transcription factor CIITA in the KO lines. CIITA is unique among transcription factors because it is tightly restricted to regulating expression of genes in the MHC II pathway (52, 53). Strikingly, RT-PCR revealed that CIITA mRNA levels were decreased in FUS, TAF15, and MATR3, but not EWSR1, KOs (Fig. 2*G*). In contrast, the MHC class I transcription regulator NLRC5 (54) was not affected in any of the KOs (Fig. 2*G*). qPCR confirmed these results (Fig. 2*H*). To further examine the role of CIITA in the ALS KOs, we carried out addback studies by transfecting the FUS KO with myc-tagged CIITA or a myc-tagged negative control plasmid. As shown in *SI Appendix*, Fig. S1, the levels of the MHC II mRNAs were significantly increased by myc-tagged CIITA, but not by the control plasmid. Thus, FUS regulates the level of expression of the MHC II pathway through the CIITA transcription factor.

### Regulation of MHC II Pathway by ALS Genes in Antigen-Presenting Cells (APCs).

To gain insight into the potential importance of the MHC II pathway in ALS, we next examined MHC II expression in APCs, considering that the main function of MHC II pathway is antigen presentation. As microglia are the main APCs in the CNS, we obtained the HMC3 microglial cell line from the American Type Culture Collection (ATCC). This line was derived from SV40-transformed embryonic microglial cells and was authenticated using several criteria including morphological characteristics, karyotyping, retention of antigenic features, and expression of the microglia marker AIF1, as well as expression of other specific microglial signature genes such as P2RY12 and TMEM119 (55).

**Fig. 3.**
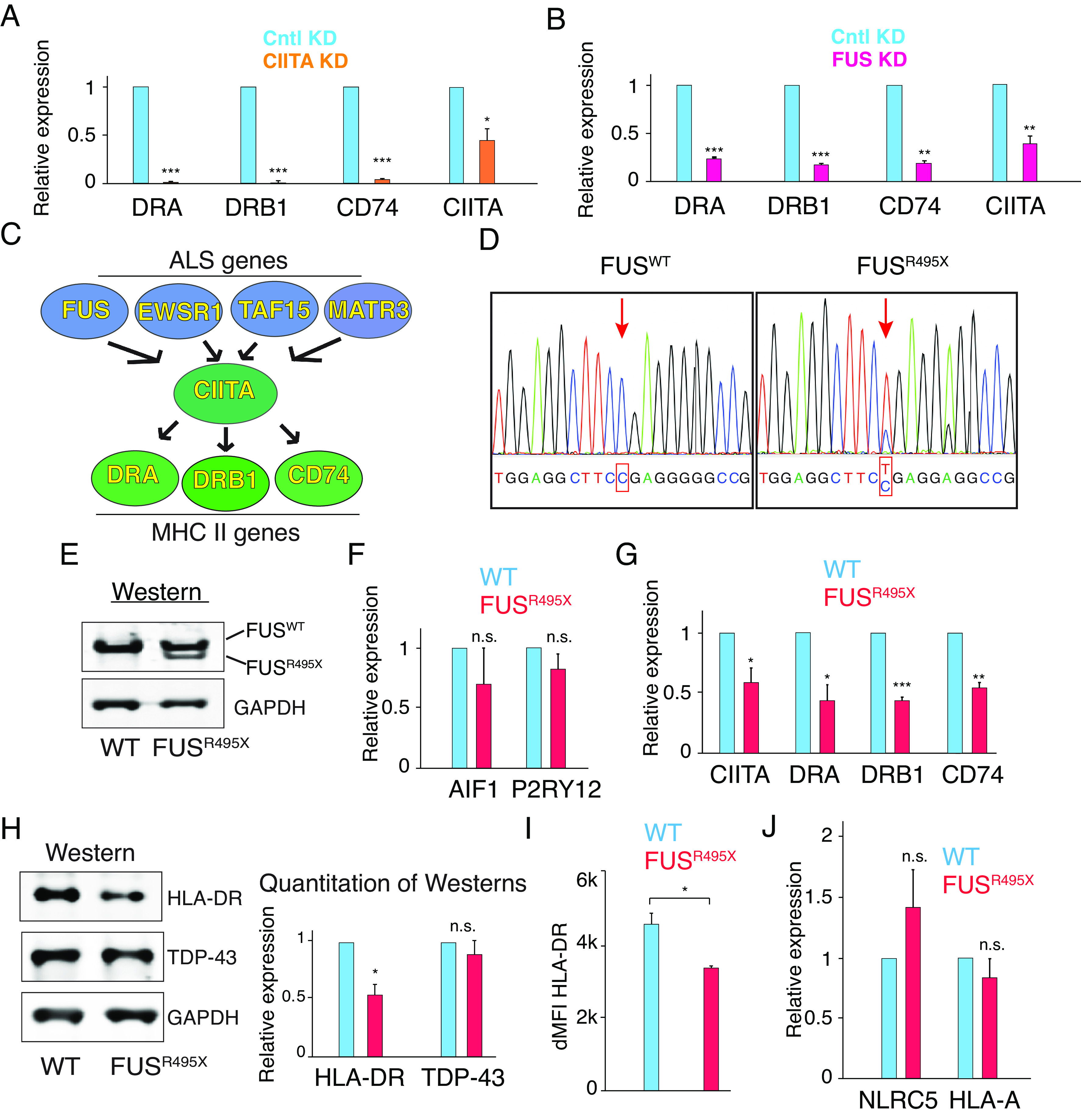
Downregulation of MHC II expression via CIITA by ALS-causative FUS mutant in HMC3 microglial line. (*A*) HMC3 cells were knocked down using CIITA siRNA or scramble (Cntl) siRNA. qPCR was carried out to examine expression level of indicated genes. *P < 0.05. **P < 0.01. ***P < 0.001. n.s., nonsignificant (paired Student’s *t* test). Error bars represent SD. (*B*) Same as *A*, except that FUS siRNA was used. (*C*) Model for regulation of MHC class II antigen presentation pathway by ALS genes. (*D*) Sanger sequencing confirmed the FUS^R495X^ heterozygous mutation introduced into HMC3 cells by CRISPR. (*E*) Westerns showing WT FUS and truncated FUS in HMC3 FUS^R495X^ cells. (*F* and *G*) Expression levels of microglia markers (AIF1 and P2RY12) and MHC II pathway genes (CIITA, HLA-DRA, HLA-DRB1, and CD74) in WT and FUS^R495X^ HMC3 cells were examined by qPCR. (*H*) Westerns using WT and FUS^R495X^ HMC3 cell lysates with antibodies against HLA-DR, TDP-43, and GAPDH. Quantitation of three biological repeats of the westerns is shown on the *Right*. (*I*) Flow cytometry was carried out using WT and FUS^R495X^ HMC cells. dMFI was calculated by subtracting median fluorescent intensity of unstained cells from that of stained cells. Three biological repeats were carried out. (*J*) Same as *G*, except that MHC I pathway genes (NLRC5 and HLA-A) were examined.

To assay the MHC II pathway in HMC3 cells, we used siRNA knockdowns (KDs) for targeting the ALS genes and CIITA. Scrambled siRNA was used as a negative control. As shown in Fig. 3*A*, CIITA KD led to greatly decreased levels of the MHC II mRNAs including both components of HLA-DR and CD74, confirming its role in expression of MHC II genes in HMC3 cells. Consistent with results obtained with HeLa cells, FUS KD in HMC3 also resulted in decreased levels of MHC II mRNAs (Fig. 3*B*). Similar results were obtained with the EWSR1, TAF15, and MATR3 KDs, whereas TDP-43 KD did not affect the expression of MHC II genes (*SI Appendix*, Fig. S2). Interestingly, the downregulation of MHC II observed in EWSR1 KD contrasts with our observation in the EWSR1 KO line. One possible explanation for this discrepancy is a genetic compensation event in the EWSR1 KO line. To validate the impact of EWSR1 on MHC II expression, we carried out an EWSR1 KD using a different siRNA. As shown in *SI Appendix*, Fig. S2*E*, levels of MHC II genes were also decreased in the EWSR1 KD by a distinct siRNA. Collectively, these results indicate that the ALS-associated proteins FUS, EWSR1, TAF15, and MATR3 all function in regulating MHC II gene expression in HMC3 microglial cells. There are several important implications of these findings regarding the MHC II pathway. As stated in the Introduction, one of the challenges in the ALS field is to understand how so many ALS-causative genes with distinct functions lead to the same disease. Our data provide an example in which multiple ALS genes regulate the same pathway (see schematic in Fig. 3*C*). Regulation of this pathway by ALS genes is especially noteworthy in a motor neuron disease, as MHC II antigen presentation pathways normally play critical roles in the CNS by protecting the neurons. Consistent with our findings, an HLA-DR polymorphism has been reported as a risk factor for ALS (56).

### Evidence Linking Downregulation of the MHC II Pathway to ALS.

Although our findings contribute an important facet to the biology of ALS genes via their role in regulating the MHC II pathway, a critical question is whether downregulation of these genes plays a role in ALS pathogenesis. To investigate this possibility, we first examined postmortem patient spinal cords. However, we observed unacceptable levels of variability in both control and ALS subjects, likely due to inherent difficulties with postmortem tissue. As another approach for determining whether the MHC II pathway is connected with ALS pathogenesis, we CRISPR-edited HMC3 cells to introduce a heterozygous FUS^R495X^ mutation. This mutation causes a severe form of the disease because most of the C-terminal NLS is truncated due to the mutation (57). Sanger sequencing and westerns showed that the R495X mutation was successfully introduced into the HMC3 cells, and the truncated R495X and WT proteins were both expressed in the mutant (MT) lines (Fig. 3 *D* and *E*, respectively). CRISPR editing of HMC3 did not affect expression of microglial markers AIF1 or P2RY12, as shown by qPCR in Fig. 3*F*. Significantly, levels of MHC II genes HLA-DRA, DRB1, and CD74, were down-regulated in the HMC3 FUS^R495X^ MT line (Fig. 3*G*). Notably, CIITA expression was also down-regulated in HMC3 FUS^R495X^ (Fig. 3*G*) whereas other MHC II-related transcription factors including RFX5, RFXAP, and RFXANP were not affected (*SI Appendix*, Fig. S3). At the protein level, approximately 50% reduction of HLA-DR was also observed in FUS^R495X^ HMC3 microglial cells (Fig. 3*H*). In contrast, there was no significant difference in TDP-43 protein levels (Fig. 3*H*). Furthermore, flow cytometry confirmed the decrease of HLA-DR protein level in FUS^R495X^ (Fig. 3*I*). Importantly, MHC I gene HLA-A and MHC I transcription regulator NLRC5 remained unchanged in FUS^R495X^ cells (Fig. 3*J*). Together, these results indicate that the ALS mutation FUS^R495X^ specifically disrupts the expression of MHC II genes via CIITA in microglial cells, providing a link between ALS and downregulation of the MHC II pathway. The pathogenesis of FUS-related ALS has been postulated to involve both loss-of-function and toxic gain-of-function mechanisms (3). Additional research is necessary to gain a better understanding of the mechanisms through which the FUS^R495X^ mutation leads to the downregulation of the MHC II pathway. Future investigations using large datasets from ALS patient samples would also shed light on the roles of different FUS mutations in MHC II expression.

### Downregulation of MHC II Pathway by ALS Mutations in HPCs.

**Fig. 4.**
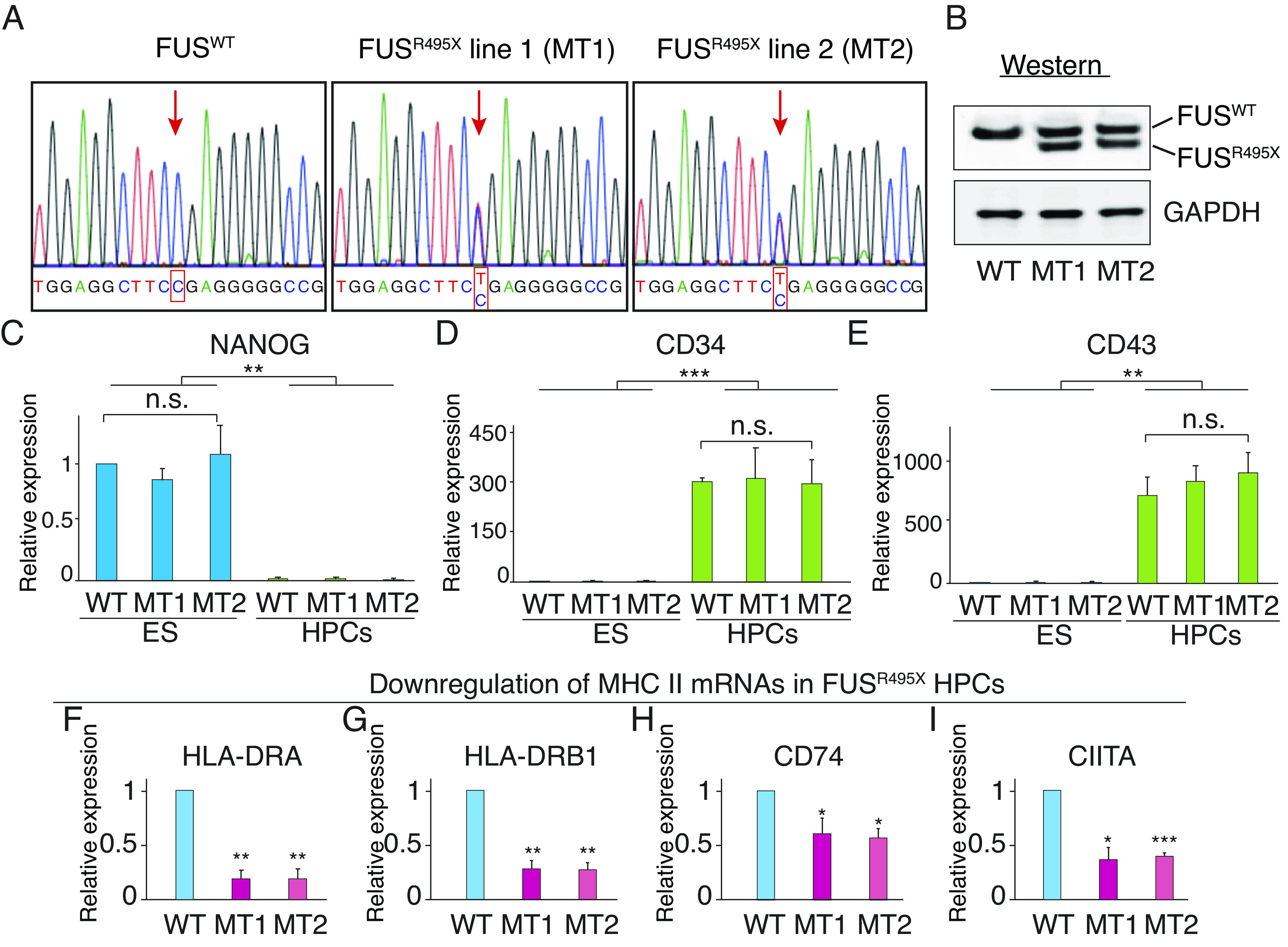
Linking downregulation of MHC II pathway to ALS. (*A*) Sanger sequencing showing heterozygous R495X FUS mutation in two independent ES lines. (*B*) Westerns showing WT FUS and truncated FUS in MT lines. (*C*–*E*) qPCRs of total RNAs from ES cells and HPCs confirming HPC differentiation. Pluripotency marker NANOG (*C*) was expressed in ES cells but not in HPCs whereas HPC markers CD34 (*D*) and CD43 (*E*) were not expressed in ES cells but were expressed in HPCs. *P < 0.05. **P < 0.01. ***P < 0.001. n.s., nonsignificant (paired student’s *t* test). Error bars represent SD. (*F*–*I*) qPCR of total RNA isolated from HPCs to assay expression levels of MHC II pathway genes.

Although the CNS is the primary tissue that is affected in ALS patients, recent studies have indicated that the general immune system plays roles in ALS disease progression (58–60). As antigen presentation is one of the most critical parts of the global immune response, we further investigated whether ALS mutations cause downregulation of the MHC II pathway in another type of immune cells. To do this, we CRISPR edited a human ES cell line to harbor the heterozygous FUS^R495X^ mutation, generating 2 independent lines that were verified by Sanger sequencing and Westerns (Fig. 4 *A* and *B*). We next differentiated the FUS^R495X^ ES cells into HPCs. We chose HPCs because they differentiate into many different MHC II cell types, including dendritic cells (DCs), macrophages, monocytes, as well as CD4+ and CD8+ T cells (61–63), many of which are involved in ALS (59, 64–67). As shown in Fig. 4 *C*–*E*, we found that both the MT and WT ES cells were efficiently differentiated into HPCs as revealed by cell-type-specific markers (NANOG as the stem cell marker, CD34 and CD43 as HPC markers). We next carried out qPCR to assay for the MHC II pathway genes. Remarkably, these data revealed a significant decrease in the levels of all the MHC II genes in the FUS^R495X^ HPCs but not in the FUS^R495X^ ES cells (Fig. 4 *F*–*I* and *SI Appendix*, Fig. S4). These findings are particularly noteworthy because of the sheer number of CNS and systemic immune cells expected to have disrupted expression of the MHC II pathway due to ALS-MT HPCs. The systemic cells enter the CNS as spinal cord infiltrates, and interactions between glial cells and the infiltrating MHC II cells are thought to be one of the mechanisms by which they contribute to ALS pathogenesis (17, 68–72). Moreover, a recent study showed that hematopoietic stem and progenitor cells (HSPCs) constitutively present antigens via MHC II, thereby activating CD4^+^ T cells and safeguarding the integrity of the HPSC pool (73). Thus, a lack of MHC II in HPCs might have profound adverse impacts on hematopoiesis and immune system development in ALS patients.

### Downregulation of MHC II Pathway Caused by C9ORF72 HRE.

**Fig. 5.**
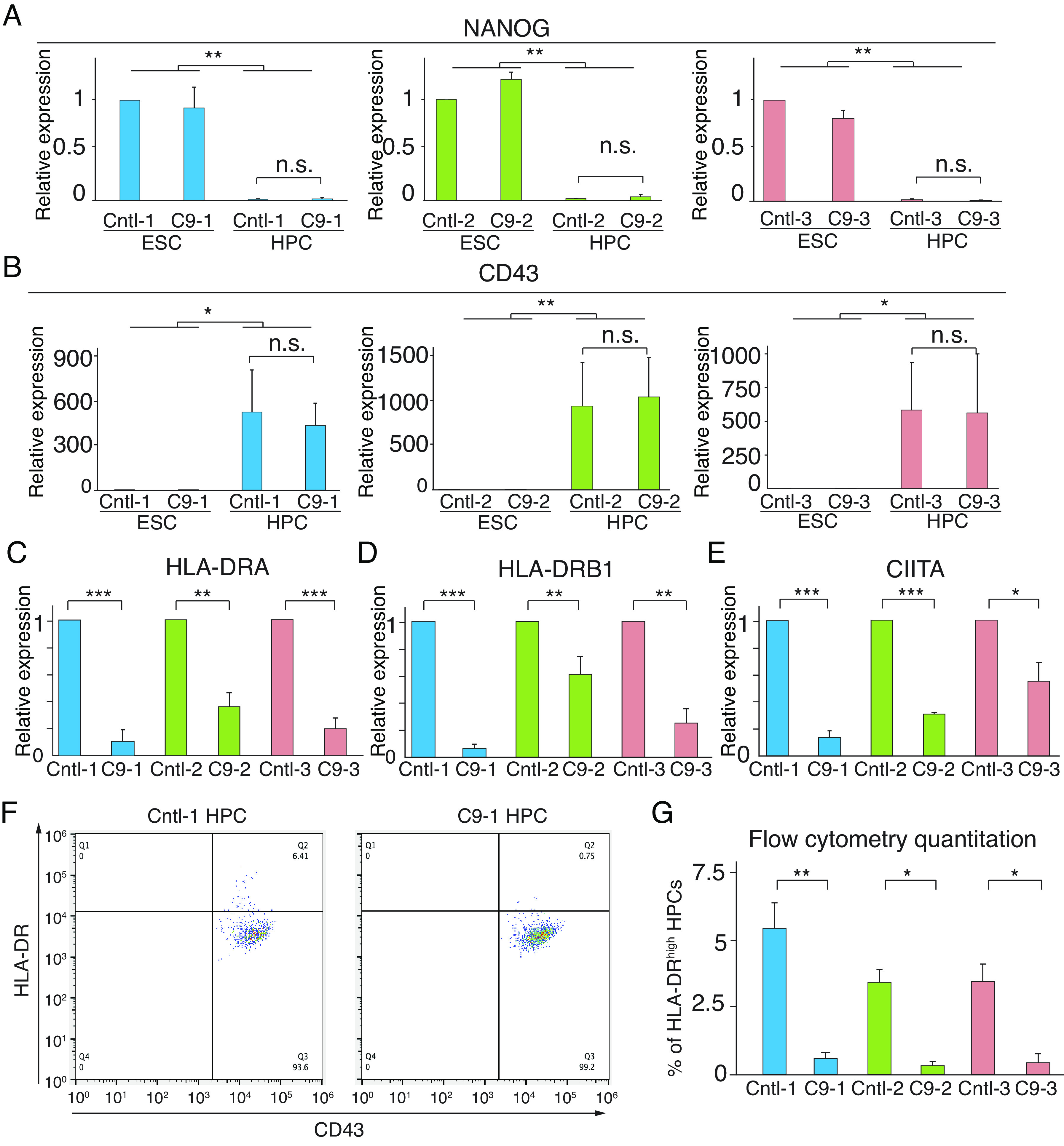
Downregulation of MHC II pathway in C9ORF72 HPCs. (*A* and *B*) Total RNAs from control (Cntl) and C9ORF72 (C9) iPSCs and HPCs were used for qPCR to confirm HPC differentiation. Pluripotency marker NANOG and HPC marker CD43 were examined. (*C*–*E*) Expression levels of indicated MHC II pathway genes were determined by qPCR using total RNAs from HPCs. (*F*) Cntl-1 and C9-1 HPCs were used for flow cytometry to examine expression of HLA-DR and CD43. (*G*) Quantitation of the percentage of HLA-DR^high^ HPCs examined by flow cytometry in 3 pairs of Cntl/C9 HPCs differentiated from iPSCs. For each pair of HPCs, three biological repeats were performed. Paired Student’s *t* test was used for statistical analysis. Error bar represents SD.

To explore whether the disruption of MHC II gene expression is a common characteristic of ALS, we investigated the effect of GGGGCC HRE in the C9ORF72 gene, which represents the most frequent genetic cause of ALS. For this purpose, we obtained 3 independent human induced pluripotent stem cell (iPSC) lines derived from three unrelated C9ORF72 ALS patients. Corresponding isogenic control iPSC lines without C9ORF72 HRE were obtained for comparison. These iPSC lines were then differentiated into HPCs following the same procedure described previously. As shown in Fig. 5 *A* and *B*, expression levels of NANOG and CD43 indicate that all iPSC lines were efficiently differentiated into HPCs, and no significant difference was observed between C9 and corresponding control iPSCs. Strikingly, MHC II molecules HLA-DRA and DRB1 were both drastically down-regulated in all three C9 HPCs, compared to the corresponding isogenic control HPCs (Fig. 5 *C* and *D*). CIITA levels were also reduced in C9 HPCs (Fig. 5*E*), similar to the results we obtained with FUS MT HPCs. The downregulation of HLA-DR protein level in C9 HPCs was also demonstrated by flow cytometry analysis. As shown in Fig. 5 *F* and *G* and *SI Appendix*, Fig. S5, all three C9 HPCs exhibited significantly fewer HLA-DR^high^ cells compared to their corresponding control HPCs. Together, these data indicate that the MHC II pathway is disrupted in HPCs carrying C9ORF72 HRE, highlighting a shared feature of MHC II dysregulation in ALS caused by mutations in FUS and C9ORF72.

In conclusion, our findings indicate that four ALS-causative proteins FUS, EWSR1, TAF15, and MATR3 have a common role in regulating MHC II expression through transcription factor CIITA. The ALS-causative R495X mutation in FUS also leads to downregulation of MHC II pathway, both in microglial cells and human ES-derived HPCs. Moreover, disruption of MHC II expression was also observed in HPCs generated by C9ORF72 ALS patients-derived iPSCs, suggesting that downregulation of MHC II is a shared feature of ALS caused by mutations in different genes. It is noteworthy that MHC II expression is also regulated by immune responses. MHC II genes have been reported to be up-regulated in several neurodegenerative diseases, including Parkinson's disease, Alzheimer’s disease, and ALS (74). Both excessive inflammation and inefficient immune responses have been observed in ALS patients, which may vary depending on the stages of the disease (17, 75). Indeed, spatiotemporal transcriptional analysis of a mouse ALS model showed significant changes in the expression level of mouse MHC II gene H2-Aa in the spinal cord at different time points (76). Furthermore, expression of MHC II can vary across different cell types. For example, in C9ORF72 knockout mice, MHC II levels were down-regulated in CD11b splenic DCs and CD8a splenic DCs but remained unchanged in plasmacytoid DCs (58). Our findings that multiple ALS genes affect MHC II expression do not exclude the involvement of other regulatory mechanisms and these mechanisms likely function simultaneously. Further investigations are needed to gain a better understanding of the spatiotemporal regulation of MHC II expression in ALS patients.

Consistent with our observation that C9 HRE led to decreased MHC II expression, C9ORF72 has been reported to be required for proper systemic as well as CNS immune responses (77, 78). Previous studies of HIV patients have shed further light on the relevance of the systemic immune system in ALS. These studies have reported a higher prevalence of ALS-like syndrome in HIV patients, which improves following anti-HIV treatment (79–83), correlating impaired immune system with ALS symptoms. Relative to the studies of motor neurons and glial cells in ALS, much less work has been done on the systemic aspect of ALS. Downregulation of the MHC II pathway by MT ALS genes in HPCs provides a plausible explanation for why ALS is not only a CNS disease but also manifests as a systemic disease. Identifying therapeutics that are efficacious at specific stages of ALS in which the CNS/systemic immune system is impacted, either positively or negatively, would be of great value for making advances in disease treatment.

## Materials and Methods

### Plasmids and Antibodies.

The myc-CIITA plasmid was obtained from Addgene (Plasmid #14650). The myc-PK plasmid was a gift from Dr. Gideon Dreyfuss (84). The GAPDH and TDP-43 antibodies were from Proteintech (Cat No. 60004-1-Ig and 10782-2-AP, respectively). The FUS antibody was described previously (27). The HLA-DR antibody was from Abcam (ab20181).

### Cell Culture.

HeLa cells were grown in Dulbecco’s Modified Eagle’s Medium (Invitrogen) plus 10% fetal bovine serum (FBS, Gibco) and 1% penicillin-streptomycin (Invitrogen). Generation of HeLa KO lines was described previously (28). HMC3 cells (ATCC^®^ CRL-3304™) were cultured in EMEM (ATCC^®^ 30-2003™) plus 10% FBS (ATCC^®^ 30-2020™) following the ATCC manual. To examine MHC II expression levels, HMC3 cells were treated with 3 ng/mL of IFNγ (Sigma) 24 h before harvest. The H9 human ES cell line was cultured using the mTeSR1 medium (STEMCELL Technologies) plus 1% penicillin-streptomycin on hESC-Qualified Matrigel (Corning)-coated tissue culture plates. All cells were cultured in a 37 °C humidified 5% CO_2_ incubator.

### siRNA KD and Plasmid Transfection.

HMC3 cells were cultured in a 6-well plate at 70% confluency for siRNA KD. ON-TARGETplus SMARTPool human siRNAs targeting FUS, EWSR1 (#1), TAF15, MATR3 (85), TDP-43, CIITA or non-targeting siRNA (Horizon Discovery) were transfected into HMC3 cells using Lipofectamine RNAiMAX (Invitrogen) following the manufacturer’s instructions. Cells were then cultured for 72 h before harvesting for RNA extraction. EWSR1 #2 siRNA is siGENOME human EWSR1 siRNA SMARTPool (Horizon Discovery).

### Whole-Cell Proteomics and GSEA.

For whole-cell proteomic analysis, WT and KO HeLa cells were cultured in 150-mm dishes. Cells were harvested at 90% confluency, and whole-cell lysates were used for quantitative mass spectrometry. Digested peptides were labeled by tandem mass tag (86) for MS3 analysis using an Orbitrap Fusion mass spectrometer coupled to a Proxeon EASY-nLC 1000 liquid chromatography pump (Thermo Fisher Scientific). GSEA analyses were performed using https://www.gsea-msigdb.org/gsea/index.jsp (version 4.0.3, gene set database C5.bp.v7.0). Pre-ranked gene lists with p-value less than 0.05 from the quantitative mass spectrometry data were used for GSEA.

### qPCR.

Total RNA was isolated from cells using Trizol (Invitrogen) according to the manufacturer’s manual. cDNA was synthesized using the UltraScript 2.0 cDNA Synthesis kit (PCR Biosystems), and qPCR was performed with gene-specific primer sets (Dataset S6) and PowerUp™ SYBR™ Green Master Mix (Applied Biosystems). All qPCR analyses were carried out on an Applied Biosystems QuantStudio 7 Flex Cycler (Thermo Fisher Scientific), and relative expression values were calculated using the comparative C_T_ method. Three biological replicates were performed for all experiments. For statistical analysis, paired Student’s *t* test was used. *P < 0.05. **P < 0.01. ***P < 0.001. n.s., nonsignificant. Error bars represent SD.

### CRISPR Editing FUS^R495X^ in ES cells.

Human ES cells (H9, WiCell Institute) were cultured in E8 medium (87) on Matrigel-coated tissue culture plates with daily medium change. The SpCas9 expression plasmid pET-Cas9-NLS-6×His (Addgene plasmid # 62933) was transformed into Rosetta™ (DE3) pLysS Competent Cells (Novagen). SpCas9 protein was purified as described (88). The sgRNA was generated using the *GeneArt Precision gRNA Synthesis Kit* (Thermo Fisher Scientific) according to the manufacturer’s instructions. To create H9 cells harboring a heterozygous R495X mutation in FUS, 0.6 μg sgRNA targeting sequence GGGACCGTGGAGGCTTCCGA was incubated with 3 μg SpCas9 protein for 10 min at room temperature and electroporated into 2 × 10^5^ H9 cells along with a ssDNA oligo (tcgtcgtggtggcagaggaggctatgatcgaggcggctaccggggccgcggcggggaccgtggaggcttcTgaggTggccggggtggtggggacagaggtggctttggccctggcaagatggattccaggtaagactttaaat). MTs were identified by Illumina MiSeq and further confirmed by Sanger sequencing and westerns. A similar method was used to create the HMC3 FUS^R495X^ line.

### iPSC Lines.

The Cntl-1 (26Z90) and C9-1 (26L6) iPSC lines were obtained from Dr. Fen-Biao Gao (89). The Cntl-2 (CS29iALS-C9n1.ISOxx), C9-2 (CS29iALS-C9nxx), Cntl-3 (CS52iALS-C9n6.ISOxx), and C9-3 iPSC (CS52iALS-C9nxx) lines were obtained from Cedars-Sinai Medical Center and were deidentified prior to use in this study.

### HPC Differentiation.

WT and MT ES lines were differentiated into HPCs using the STEMdiff Hematopoietic Kit (STEMCELL Technologies). To prepare for differentiation, ES cells were detached using ReLeSR (STEMCELL Technologies) and plated on Matrigel-coated 6-well plates to achieve ~40 attached colonies/well 24 h after seeding. Medium was changed according to the manufacturer’s manual. HPCs were harvested on day 12 for RNA extraction or flow cytometry.

### Flow Cytometry.

Cells were washed with ice-cold PBS (phosphate buffered saline) and incubated with Fc Receptor Binding Inhibitor Antibody (Thermo Scientific) according to the manufacturer’s instructions. Cells were then suspended in 500 mL of flow cytometry buffer (10% PBS, 1% Sodium Azide in PBS) and stained with 5 mL of FITC-conjugated HLA-DR antibody (Clone LN3, STEMCELL Technologies) and 2.5 mL of PE-conjugated CD43 antibody (Clone 10G7, STEMCELL Technologies) for 1 h. Flow cytometry was carried out by an Attune NxT Flow Cytometer (Thermo Scientific), and data were analyzed using FlowJo software. For statistical analysis, paired Student’s *t* test was used. *P < 0.05. **P < 0.01. ***P < 0.001. n.s., nonsignificant. Error bars represent SD.

## Supplementary Material

Appendix 01 (PDF)

Dataset S01 (XLSX)

Dataset S02 (XLSX)

Dataset S03 (XLSX)

Dataset S04 (XLSX)

Dataset S05 (XLSX)

Dataset S06 (XLSX)

## Data Availability

All study data are included in the article and/or supporting information.
