# Supplementary material for: Causal ALS genes impact the MHC class II antigen presentation pathway
Source: Proc Natl Acad Sci U S A. 2023 Sep 18;120(39):e2305756120. doi: 10.1073/pnas.2305756120 (PMC10523463; doi:10.1073/pnas.2305756120)
Supplement: Supplementary file 1 — Appendix 01 (PDF) [file pnas.2305756120.sapp.pdf]

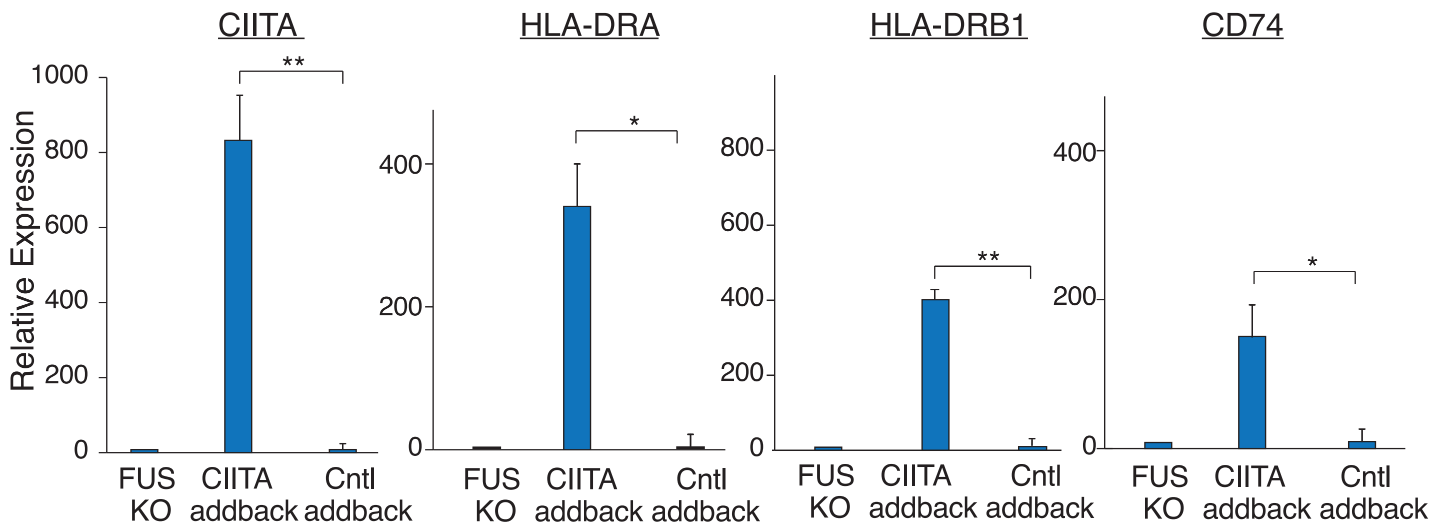

Supplementary Figure S1. Addback of CIITA to FUS KO restores expression of MHC II genes. Myc-CIITA or myc-PK (Cntl) plasmid was transfected into FUS KO HeLa cells cultured in 6-well plates (10 ng/well). Total RNAs were extracted after 24 hr and qPCR was carried out to examine expression level of indicated genes.

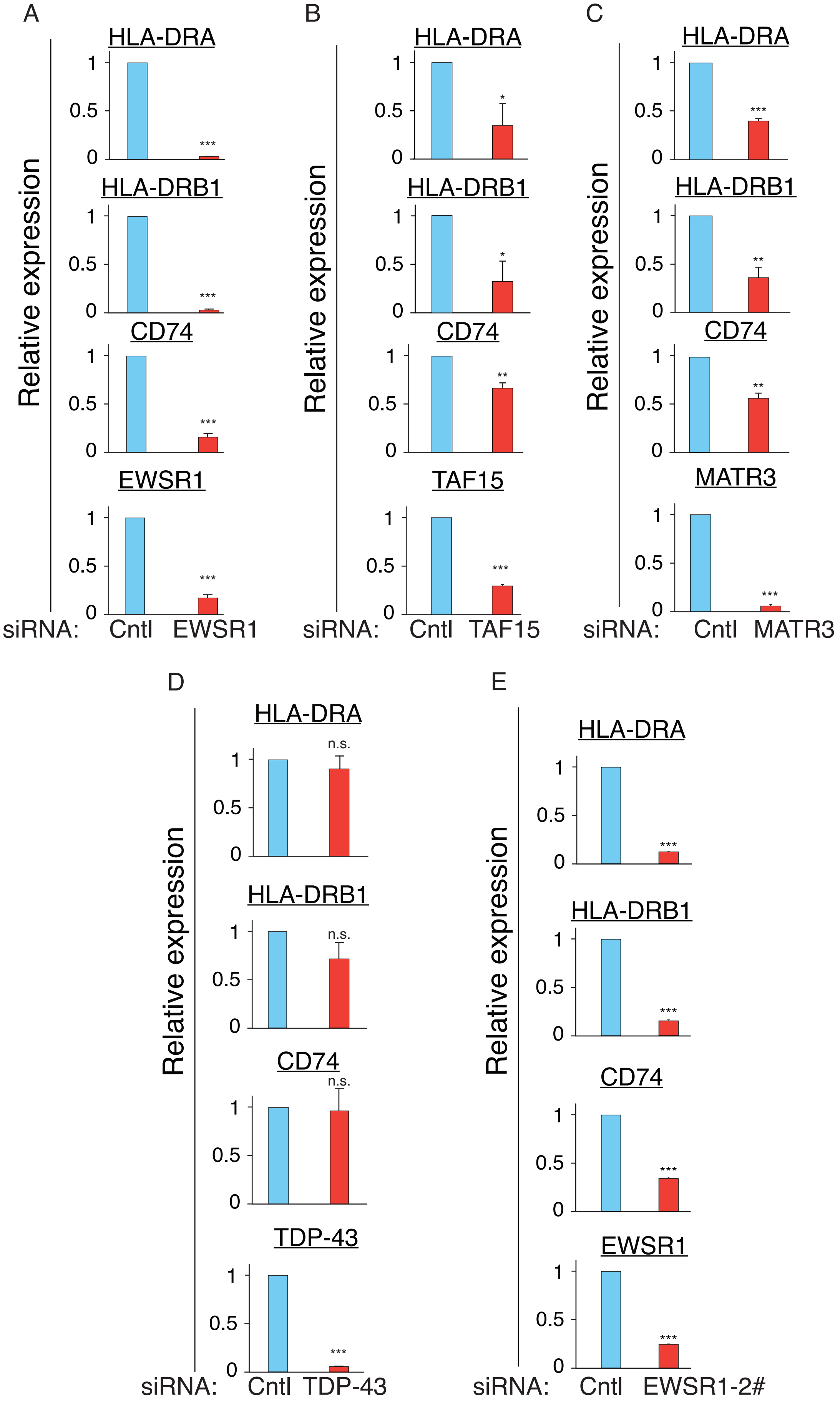

Supplementary Figure S2. MHC II pathway is downregulated in EWSR1, TAF15, or MATR3 KD HMC3 cells. EWSR1 (A), TAF15 (B), MATR3 (C), or TDP-43 (D) siRNA was transfected into HMC3 cells. Total RNAs were extracted 72 hr after transfection and qPCR was performed to examine expression level of indicated genes. (E) Same as A, except that a different EWSR1 siRNA was used.

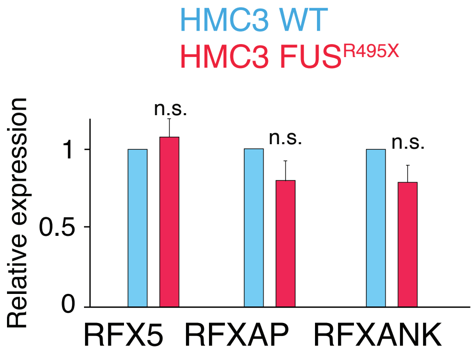

Supplementary Figure S3. ALS-causative FUS mutant in HMC3 does not affect expression level of RFX5, RFXAP, or RFXANK. Total RNAs of WT and FUS MT HMC3 lines were used for qPCR to examine expression levels of indicated transcription factors.

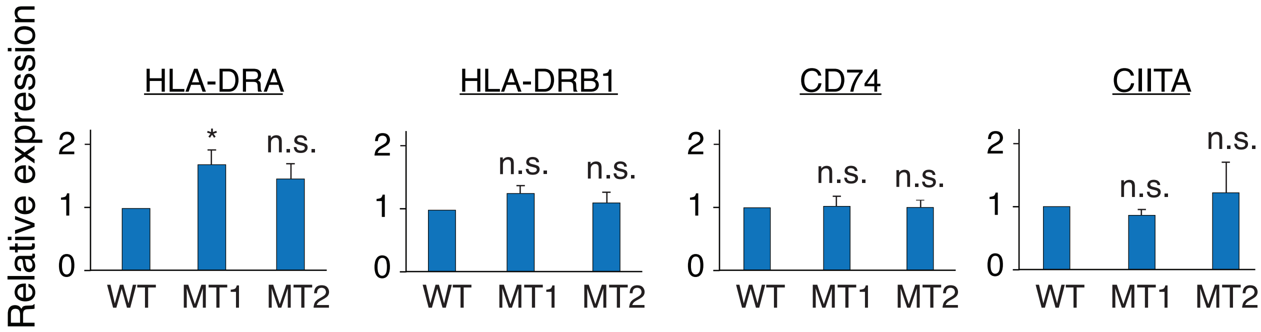

Supplementary Figure S4. MHC II pathway is unaffected in FUS MT ES lines.
Total RNAs of WT and FUS MT ES lines were used for qPCR to examine expression levels of MHC II pathway proteins.

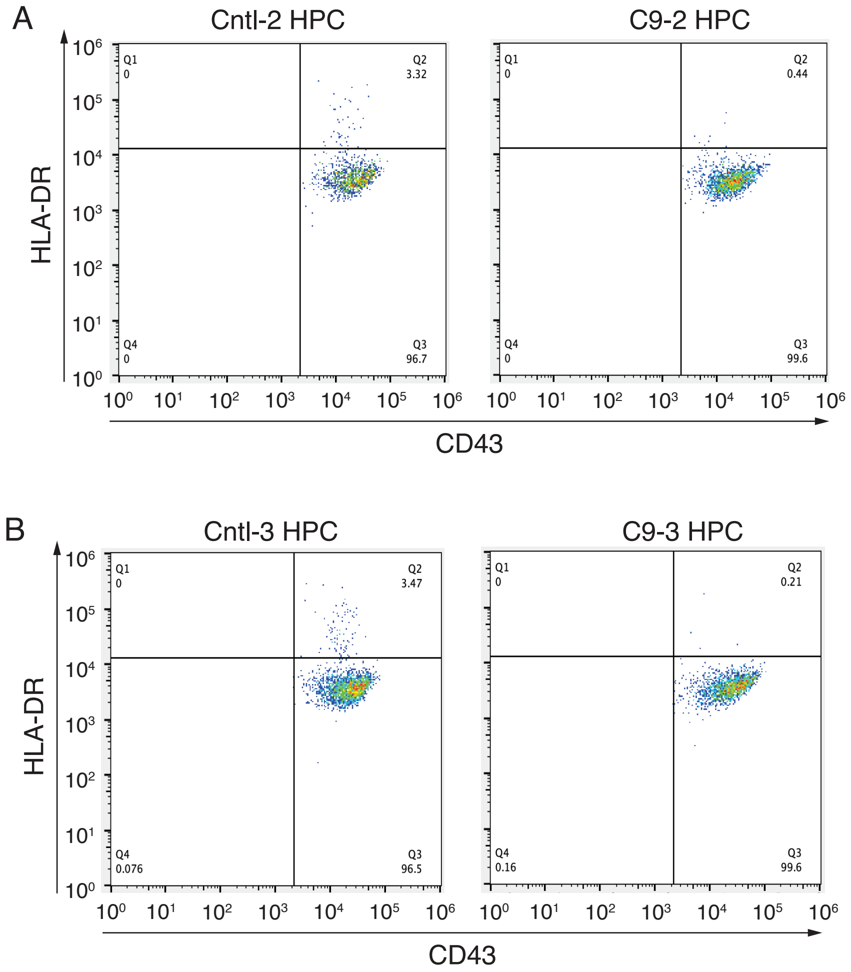

Supplementary Figure S5. Flow cytometry of HPCs differentiated from additional C9 iPSC lines. Cntl-2/C9-2 HPCs (A) and Cntl-3/C9-3 (B) were used for flow cytometry to examine expression of HLA-DR and CD43. Quantitation of three biological repeats is shown in Figure 5G.
